# Top Three Learning Platforms for Orthopaedic In-Training Knowledge Produce Different Results

**DOI:** 10.5435/JAAOSGlobal-D-21-00148

**Published:** 2021-08-03

**Authors:** Adam Margalit, Patrick Mixa, Louis Day, Majd Marrache, Stuart Mitchell, Krishna V. Suresh, Kevin Wang, Samir Sabharwal, Tuo Peter Li, Alexander Loeb, Qais Naziri, R. Frank Henn, Dawn Laporte

**Affiliations:** From the Department of Orthopaedic Surgery, Johns Hopkins University School of Medicine (Dr. Margalit, Dr. Marrache, Dr. Mitchell, Suresh, Wang, Dr. Sabharwal, Dr. Li, Dr. Loeb, Dr. Laporte); the Department of Orthopaedic Surgery, University of Maryland School Medicine, Baltimore, MD (Dr. Mixa, Dr. Hen III); and the Department of Orthopaedic Surgery, SUNY Downstate Health Sciences University, Brooklyn, NY (Dr. Day, Dr. Naziri).

## Abstract

To compare the influence of objective and subjective measures of the three learning programs (OrthoBullets [OB], ResStudy [RS], and Clinical Classroom [CC]) on resident test performance and study platform preference.

**Methods:**

Sixty residents from three orthopaedic residencies were included in this study during May 2020. Trauma, pediatrics, and hip/knee reconstruction (joints) were chosen as testing topics. Residents took a standardized pretest of 30 questions per topic, followed by the completion of 50 questions per day for 5 days using one of the three web-based programs, followed by a standardized subject-specific posttest. This cycle was repeated for all the three topics. Bivariate statistics and a mixed-effects linear regression model were used to compare improvements in the scores.

**Results:**

Across all learning platforms, topics, and postgraduate year classes, posttest scores were 4.4% higher than the pretest score (73.3% vs. 68.9%, *P* < 0.001): 6.8% higher with OB, 5.4% with RS, and 1.0% with CC. The score improvement with OB was significantly greater than the score improvement with CC (*P* < 0.001). In total, 100% of residents reported that using OB would improve their score on the orthopaedic in-training examination, compared with 95% with RS and 67% with CC.

**Conclusion:**

OB demonstrated the greatest improvement in scores, followed closely by RS and then CC.

For graduating residents in the United States, board certification examinations are very important and are heavily regulated to ensure that competency standards are maintained.^1^ After orthopaedic surgery training, residents must pass both the written and the oral part of the American Board of Orthopaedic Surgery (ABOS) examination to be certified as diplomats of the ABOS. Because the Accreditation Council for Graduate Medical Education monitors every residency program's board pass rate, programs have a vested interest in identifying tools to track resident education progress and promote effective learning strategies.^2-4^

Currently, the orthopaedic in-training examination (OITE) is the primary objective assessment of resident knowledge throughout training and is highly predictive of performance on ABOS part I written examination.^2^ Established in 1963 by the American Academy of Orthopaedic Surgeons (AAOS), the OITE is administered annually and serves as a tool that allows programs to assess their residents and identify individuals at risk of failing the ABOS examination.^5^ Over the last decade, several online question banks—including Clinical Classroom (CC), OrthoBullets (OB), and ResStudy (RS)—have been developed with the ultimate goal of promoting long-term information retention through reinforced cycles of information processing and retrieval.^6-8^ The relative utility of each of these resources in improving OITE test scores among orthopaedic residents has not been investigated.

Identification of optimal learning strategies and preparation resources available to orthopaedic surgery residents can improve their performance on the OITE, which may translate to superior clinical performance.^9^ The primary purpose of this study was to compare objective measures of the top three learning web-based programs OB, RS, and CC for OITE performance. A secondary objective of this study was to use a survey to determine subjective resident preference of these three learning platforms. We hypothesized that, objectively, all three web-based programs would increase a resident's score and that, subjectively, there would be an equal distribution of residents preferring each study platform.

## Methods

### Study Population, Setting, and Design

This was a prospective cohort study of resident orthopaedic surgeons from three US allopathic orthopaedic residences during May 2020. Residents in postgraduate years (PGY) 1 to 4 were included in our analysis. PGY-5 residents were excluded from our study design because of concurrent preparation for ABOS part I written examination during the period in which this study was conducted. Because each residency program had six residents per PGY class, a total of 72 residents met the initial inclusion criteria.

Residents were divided into three separate groups (labeled A, B, and C), each composed of members from all included PGY classes. All groups were assigned to a 15-day study schedule, which was subdivided into 5-day blocks corresponding to one of the three specific OITE topics. Each block consisted of a 30-question pretest on a single topic, followed by 50 questions completed daily on an assigned learning platform, and finally a 30-question posttest at the conclusion of each block (Table 1). Groups differed in the type of learning platform used for a given OITE topic (Table 2). Group study was not permitted, and residents were only allowed to use the specific learning platforms assigned to them during that block. If residents were unable to complete those 50 questions daily, they were allowed to catch up on subsequent days as long as 250 questions were completed by the end of each 5-day block. Performance on each pretest and posttest examination was collected for all participants and recorded for the percentage of questions answered correctly. At the conclusion of the 15-day study period, residents were asked to complete a questionnaire evaluating their experience with each learning platform.

**Table 1:** 15-Day Study Plan

Block 1—Trauma	Day 1	Day 2	Day 3	Day 4	Day 5
	Pretest trauma (30q)	50 questions platform-specific	50 questions platform-specific	50 questions platform-specific	50 questions platform-specific, followed by posttest trauma (30q)
	50 questions platform-specific				
Block 2—Pediatrics	Day 6	Day 7	Day 8	Day 9	Day 10
	Pretest pediatrics (30q)	50 questions platform-specific	50 questions platform-specific	50 questions platform-specific	50 questions platform-specific, followed by posttest pediatrics (30q)
	50 questions platform-specific				
Block 3—Joints	Day 11	Day 12	Day 13	Day 14	Day 15
	Pretest joints (30q)	50 questions platform-specific	50 questions platform-specific	50 questions platform-specific	50 questions platform-specific, followed by posttest joints (30q)
	50 questions platform-specific				Poststudy completion survey

**Table 2:** Learning Platform Assigned per OITE Topic for Each Resident Group

	Trauma	Pediatric	Joints
Group A	OB	RS	CC
Group B	RS	CC	OB
Group C	CC	OB	RS

CC = Clinical Classroom, OB = OrthoBullets, OITE = orthopaedic in-training examination, RS = ResStudy

Of the 72 residents who initially met the inclusion criteria, 39 (54%) completed the study plan in its entirety, 12 (17%) residents completed two platforms, 9 (13%) residents completed only one platform, and 12 (17%) residents completed none of the platforms. After excluding the residents who completed none of the study platforms, a total of 60 residents and 300 test scores (150 pretests and 150 posttests) were included in our final cohort.

### Topics

Three topics were chosen for this study: trauma, pediatrics, and joints. These topics represent the most highly tested material on OITEs from 2015 to 2019 (Table 3). Shoulder was not selected as a topic because it is not consistent in the top three represented topics from 2015 to 2019. Although the inclusion of basic science was considered, these questions were grouped with oncology on the CC learning platform. Because of the inability to isolate questions across all learning platforms, this subject was excluded from our study design.

**Table 3:** 2015 to 2019 OITE Subject Material Percentage Breakdown

	2015	2016	2017	2018	2019	Average
Total	266	269	271	269	259	267
Trauma	18%	18%	18%	17%	12%	15%^a^
Sports	8%	9%	8%	3%	7%	7%
Spine	9%	7%	7%	7%	12%	10%^a^
Shoulder	9%	7%	8%	11%	15%	12%
Practice management	—	—	2%	2%	1%	1%
Pediatrics	13%	13%	12%	11%	7%	10%^a^
Oncology	8%	8%	8%	8%	8%	8%
Joints	8%	10%	10%	13%	11%	10%^a^
Hand	7%	8%	7%	7%	8%	7%
Foot and ankle	8%	7%	7%	8%	7%	8%
Basic science	12%	12%	12%	12%	12%	12%^a^

OITE = orthopedic in-training examination

a Denotes subject matter that are heavily represented in OITE.

### Learning Platforms

Three learning platforms were evaluated in this study as follows: OB, RS, and CC. OB was developed in 2011 and currently has over 7,000 questions available from multiple sources including 2007 to 2013 AAOS self-assessment examination (SAE) questions, OITE-based questions (OBQ), and SAE-based questions (SBQ). OBQ and SBQ are generated internally from the OB authors.^10^ The OB authors typically consist of resident physicians who achieved at least one >90th percentile OITE scores based on PGY class. Each question undergoes a rigorous peer-review process consisting of two independent reviewers with multiple stages of edits and strict tested content criteria.^10^ The review content is multimodal and includes case-based learning, videos, operative techniques, and discussion forums. OB includes a curriculum across various orthopaedic topics, with the ability to track daily progress through the material and a proprietary machine learning–enabled spaced repetition algorithm, called Anconeus, which provides users with daily learning question reinforcement.^10^

RS was introduced in 2012 and has approximately 5,000 questions available for users. These questions are taken from previous 2012 to 2019 OITEs, 2015 to 2020 SAE questions, and previous Orthopaedic Knowledge Update examinations. Similar to OB, RS also offers residents the ability to select topics for testing and the ability to track the examination history and progress but does not offer a comprehensively structured educational curriculum.^11^ However, RS typically explains the rationale for answer choices by citing previously published literature to adequately explain the tested concept.

Finally, CC was developed in 2017 and uses adaptive algorithms to reinforce learning based on user input regarding the perceived difficulty level for each question. After every answered question, users rank their subjective level of confidence with one of the four labels: (1) know it, (2) think so, (3) unsure, and (4) no idea. Using this information, proprietary adaptive learning algorithms generate question sets that continually test areas of weakness. CC has approximately 3,500 questions, all generated from well-known experts across various orthopaedic subspecialties. Similar to OB and RS, CC offers users the ability to filter questions by topic.^12^ Although the residents participating in this study had access to OB and RS for several years, access to CC was obtained just before this study.

### Pretests and Posttests

Sixty questions for each topic (trauma, pediatrics, and joints) were randomly selected from the 2007 to 2013 AAOS SAE examinations found on the OB question database. Each set of 60 questions was divided equally into two sets, a pretest and a posttest. Question difficulty between the pretests and posttests was matched for each set of examinations based on the average percentage of OB users who answered a given question correctly. Considering the large sample size of OB users per question, this method served as a proxy for question difficulty. For participants who used OB as a learning platform, 2007 to 2013 SAE questions were deselected from the question bank in all user accounts so that all practice questions only consisted of OB generated OBQ and SBQ only. This way, we ensured that all pretest and posttest questions were curated from previous subject-specific SAE which were not available in any of the three web-based learning platforms and that no learning platform offered residents an unfair advantage. One PGY-5 resident (S.M.) reviewed the pretest and posttest questions to ensure that the most testable and appropriate topics were included.

**Table 4:** Pretest and Posttest Percentages by Study Platform

	N (tests)	Pretest percentage	Posttest percentage	Difference, %
Total	150	68.9	73.3	4.4
OrthoBullets	50	70.0	76.8	6.8
Clinical Classroom	49	69.7	70.7	1.0
ResStudy	51	67.1	72.5	5.4

### Survey Design

An anonymous 22-question survey was developed based on surveys used in similar studies and constructed in classmarker.com (Appendix I, http://links.lww.com/JG9/A150).^13-15^ The response data were downloaded directly from the website. All residents who completed the study were given the option to complete this survey. Participation in the survey was voluntary. The questions assessed resident perception of each learning platform's quality, efficacy, and relevance in relation to the in-training examination. These questions also assess what kinds of learning strategy residents were used to study for the in-training examination in the past and what they prefer to use in the future if given limited resources.

### Statistical Analysis

A power analysis was done demonstrating that 150 pretests and 150 posttests were needed to detect a 5% difference between pretest and posttest scores with 80% of power. For bivariate analyses, independent samples' *t*-test, paired samples' *t*-test, and one-way analysis of variance with post hoc analyses were done where appropriate. Because some residents only completed one or two of the platforms while others completed all three, we incorporated a mixed-effects generalized linear regression model to control for (1) inherent differences in test-taking ability between residents, (2) different pretest scores between PGY-classes, and (3) differences in percentages across the study topics. Based on our study design, test scores were nonindependent observations because each resident could account for more than one test score in different topics or learning platforms. In other words, test scores from all three study platforms and all three topics were compiled from tests taken by the same cohort of residents, making these test scores nonindependent of each other. As such, a mixed-effects model was chosen to account for both fixed and random effects stemming from the nonindependence within our data set that was inherent to our study design. Statistical significance was set at *P* = 0.05. Statistical analyses were done with the assistance of a statistician using STATA version 15.0 (StataCorp, College Station, TX).

## Results

### Change in Test Scores by Learning Platform and Topic

**Figure 1:**
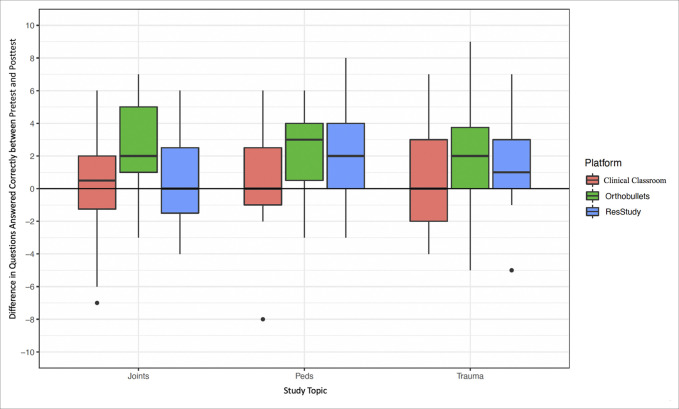
Graph demonstrating improvement in questions answered correctly by the study platform and the study topic.

Across all learning platforms, topics, and PGY classes, posttest scores were 4.4% higher than pretest scores (73.3% vs. 68.9%, *P* < 0.001; Figure 1 and Table 4). Scores improved by 6.8% with OB, 5.4% with RS, and 1.0% with CC. After controlling for pretest score, PGY class, and resident in the mixed-effects regression, the score improvement with OB was significantly greater than the score improvement with CC (*P* < 0.001), but there were no significant differences in comparison between any other study platforms (Table 5). For additional questions answered correctly, completing 50 questions daily for 5 days on any platform resulted in approximately one additional correct answer on the posttest.

**Table 5:** Mixed-Effects Generalized Linear Regression Model

	Additional Correct Questions	95% CI	*P* value
Study platform			
Clinical Classroom			Referent
OrthoBullets	0.32	0.15–0.49	**<0.001**
ResStudy	0.16	0.15–0.49	0.057
PGY class			
PGY-1			Referent
PGY-2	0.17	0.15–0.49	0.170
PGY-3	0.43	0.15–0.49	**0.001**
PGY-4	0.52	0.15–0.49	**<0.001**

PGY = postgraduate year

Bold values indicate statistical significance.

**Figure 2:**
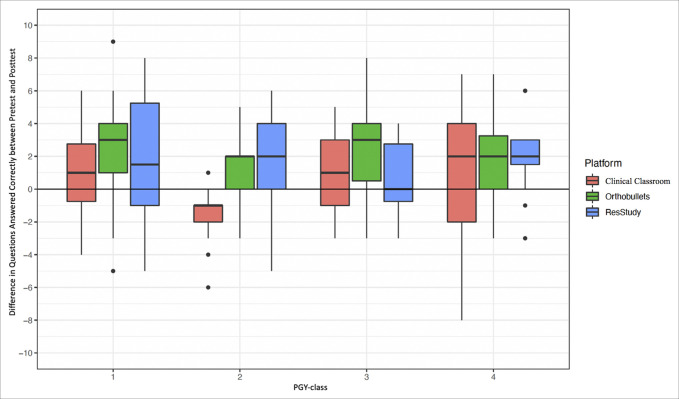
Graph demonstrating improvement in questions answered correctly by the study platform and PGY class. PGY = postgraduate year.

Across all study platforms, test scores increased the most for pediatrics (5.5%), followed by trauma (4.3%) and joints (2.9%) (Table 6 and Figure 2). Relative to OB user averages, residents scored 2.2% less on pretests and 2.7% higher on posttests across all topics (*P* = 0.012; Table 6).

**Table 6:** Percentage Improvement in Scores by Study Topic

		OB User Average		Study Cohort		Study Cohort Relative to Nation		Improvement Relative to Nation	*P* value
	N (tests)	Pretest	Posttest	Pretest	Posttest	Pretest	Posttest		
Total	150	71.1%	71.2%	69.0%	72.9%	−2.1%	1.6%	3.7%	**0.012**
Trauma	59	72.0%	70.9%	68.3%	71.7%	−3.7%	0.8%	4.5%	**0.049**
Pediatrics	51	70.4%	71.4%	71.2%	77.7%	0.8%	6.3%	5.5%	**0.007**
Joints	40	70.5%	71.5%	67.1%	68.4%	−3.4%	−3.1%	0.3%	0.459

OB = OrthoBullets

Bold values indicate statistical significance.

### Change in Test Scores by Postgraduate Year Class

On unadjusted analysis, there were no significant differences in score improvement between the PGY classes across all study platforms (6.0% improvement for PGY-1, 1.1% for PGY-2, 4.8% for PGY-3, and 5.3% for PGY-4; *P* > 0.05; Table 7 and Figure 2). However, because PGY-1 residents had significantly lower pretest scores than all other PGY classes (*P* < 0.001, Table 7), adjusted analysis was done to more robustly assess score improvement. After controlling for differences in pretest scores and study topic and resident in the mixed-effects regression, PGY-3 and PGY-4 residents demonstrated significantly greater improvement in test scores compared with PGY-1 residents (*P* < 0.001 for both; Table 5). There remained no significant differences in score improvements between PGY-2 and PGY-1 residents on adjusted analysis (*P* > 0.05).

**Table 7:** Pretest and Posttest Percentages by PGY Class

	N (Tests)	Pretest Percentage	Posttest Percentage	Difference, %
Total	150	68.9	73.3	4.4
PGY-1	45	57.0	63.0	6.0
PGY-2	37	72.6	73.7	1.1
PGY-3	31	74.2	79.0	4.8
PGY-4	37	75.3	80.6	5.3

PGY = postgraduate year

### Survey Results

At the completion of the study, 32% of all residents (23) responded to the survey. When asked to choose a single study resource, 52% (12) of residents chose OB, 48% (11) chose RS, and 0% (0) chose CC. On average, residents rated OB as 9 of 10, RS as 8 of 10, and CC as 5 of 10. In total, 100% of residents (23) thought that studying using OB would improve their score on the OITE, compared with 96% (22) with RS and 65% (15) with CC. When asked to rank the ease of use, 52% of residents (11) ranked OB first, compared with 43% (10) with RS and 5% (1) with CC (Supplementary Table 1, http://links.lww.com/JG9/A149).

Free response resident feedback was collected to comprehensively evaluate the likes and dislikes of each learning platform (Supplementary Table 2, http://links.lww.com/JG9/A149). Positive comments for each learning platform included “OB has best explanations and well-designed Anconeus spaced repetition tool,” “ResStudy has the most recent OITE questions and is most similar to OITEs,” and “CC has multiple types of questions, not just multiple choice, along with their Recharge spaced repetition tool.” Criticism of each learning platform included “OB only has older SAE questions and no OITE questions,” “ResStudy has no repetition learning and does not provide explanations for all questions,” and “CC is not similar to OITE questions and has limited number of questions compared with other platforms.”

Residents were also asked to provide preferred learning platforms and additional study resources used to prepare for the 2019 OITE (Supplementary Table 3, http://links.lww.com/JG9/A149).

## Discussion

Evaluating current learning resources available for OITE preparation can assist residents in choosing the best study products to improve their OITE scores, which have been shown to be highly predictive of ABOS part I written examination.^2^ The goal of this study was to rigorously evaluate and compare the effectiveness of three popular online learning platforms for improving OITE scores across multiple residency programs. We found that completing 50 questions daily across a 5-day study period for a given topic resulted in markedly improved scores, regardless of the learning platform, and that OB was associated with markedly greater score improvements compared with CC after adjusting for resident, PGY class, and pretest scores. RS was associated with less score improvement than OB, but greater improvements than CC, although these differences did not reach statistical significance.

These results support several important implications for both orthopaedic residents and residency programs. From a resident perspective, choosing the study platform and strategy that is most likely to result in a higher OITE score not only may improve a resident's orthopaedic knowledge but also saves time by eliminating unnecessary efforts spent on studying less impactful resources. From a residency program perspective, OITE performance is highly important to residency programs because an overall pass rate of >80% (over 3 years) within a given program is required to maintain program Accreditation Council for Graduate Medical Education.^2-4^ Furthermore, many residency programs pay for one study platform for their residents, and although the costs of these learning platforms are similar, residency programs may be able to garner a greater return on their investment by purchasing the most effective learning resource.

The differences in effectiveness between the three study platforms may be explained by how well each study platform's practice question approximates OITE questions. CC, which was associated with the lowest score improvement, contains questions derived from panels of experts, rather than from previous OITEs.^12^ In our survey, residents noted this difference between CC questions and OITE questions. In contrast to CC, RS uses questions from recent OITEs and SAEs and is thus more representative of an OITE for the question style and content.^11^ Although OB does not incorporate any actual previous OITE questions, OB offers OBQ and SBQ questions which are specifically designed by the OB authors to mirror OITE questions.^10^ Because our posttests were generated from a random sample of 2007 to 2013 SAE questions, CC questions may not have approximated standardized exam questions as accurately as OB or RS, leading to poorer posttest performances.

All three study platforms included in our study used practice questions as the primary study strategy, which has been shown to be effective for long-term information retention.^8^ In a randomized controlled trial of 65 pediatric and emergency medicine residents, Larsen et al.^6^ demonstrated that study participants who focused on practice questions had markedly greater long-term retention of information than those who passively reviewed the material. However, the way in which practice questions are incorporated into a learning style can add to the effectiveness of any learning strategy and may explain, in part, why the largest score improvement was seen in OB. OB uses a proprietary repetition algorithm which continually tests areas of weakness in a systematic way that promotes active recall and long-term retention. In several studies, this type of “spaced-repetition” has been demonstrated to reinforce neural patterns and improve recall.^12^ CC also incorporates an automated repetition algorithm that serves to improve long-term retention. RS does not currently include a repetition algorithm, which was noted as a shortcoming of the platform based on survey comments. Ultimately, it is likely that both the similarity of OB practice questions to OITE questions and the robustness of its Anconeus spaced-repetition algorithm collectively contributed to its effectiveness and preference as a study tool compared with CC in our resident population.

The results of this study must be interpreted in context of its limitations. First, our pretest and posttest questions were derived from OB 2007 to 2013 SAE questions, and although there was no overlap of years with the 2015 to 2020 SAE questions on RS, we cannot be certain that some older SAE questions did not appear on the newer forms, although this situation would be highly unlikely. Moreover, the relatively small sample size of 250 questions per subject topic may limit the significance of our results; however, because of time constraints of didactic learning in the pandemic, we were unable to ask residents to complete more than 50 questions daily. Extraneous testing factors—such as location and concurrent work schedule—may have affected resident performance on pretests and posttests and were not accounted for in our study design. It is also possible that residents' lack of familiarity with CC relative to OB and RS may also have contributed to the findings. In addition, it is important to note that individuals have unique learning styles and may benefit differently from various question-based study platforms. Although question-based study platforms were tested, it is still critical for residents to use other resources for a solid core knowledge base. However, in our study, OB resulted in the best posttest improvement that does not necessarily correlate with a better comprehensive knowledge base. CC promotes using a metacognitive approach to improve critical thinking and complex decision making, with a focus on mastery and reinforcement more so than answering test questions. This overall study design was particularly unique because it involved residents from three different residency programs across a span of 2 weeks. A major factor as to why this was accomplished in May 2020 was because of the cancellation of elective cases and adoption of a modified resident schedule during the first wave of coronavirus disease 2019.^16,17^ Because of stay-at-home orders during the pandemic, we were unable to strictly enforce the completion of practice questions across learning platforms or the poststudy questionnaire among our resident cohort. As such, only 39 of 60 (65%) of residents completed the study plan in its entirety, and 23 of 60 (32%) completed the poststudy questionnaire, which may limit our ability to draw notable conclusions from the data set. Moreover, although we requested residents to use their assigned learning platform, we were unable to verify this during each block of the 2-week study period. Finally, because participants were aware that they were being observed during the study period, they were susceptible to the Hawthorne effect, which may have influenced their performance on pretests and posttests. Additional investigation focusing on the effect on actual OITE and/or ABOS part I scores in relation to each platform may be valuable. Additional studies may also evaluate which learning platforms or components have the greatest effect on establishing a foundational knowledge base and potentially superior clinical performance.

## Conclusion

OB demonstrated the greatest improvement in scores, followed by RS and then CC. These results provide useful information and insights for residents and residency programs seeking optimal learning resources for OITE preparation.

## Supplementary Material

SUPPLEMENTARY MATERIAL
