# Supplementary material for: Top Three Learning Platforms for Orthopaedic In-Training Knowledge Produce Different Results
Source: J Am Acad Orthop Surg Glob Res Rev. 2021 Aug 3;5(8):e21.00148. doi: 10.5435/JAAOSGlobal-D-21-00148 (PMC8337059; doi:10.5435/JAAOSGlobal-D-21-00148)
Supplement: SUPPLEMENTARY MATERIAL [file jagrr-5-e21.00148-s001.docx]

**Supplementary Tables**

**Supplementary Table 1:** *Survey questions 1-11:* Summary of survey question responses demonstrating resident evaluation of each platform’s ability to result in improved knowledge base, pre-and post-test question quality, and preferences for a single learning platform. **Appendix I** lists all survey questions and possible responses. *Abbreviation: OB=Orthobullets, RS= ResStudy, CC=JBJS Clinical Classroom, OITE=Orthopaedic In-training examination*

| **Survey Question** | **Survey Response** |
| --- | --- |
| Q1) As a result of completing ResStudy, do you feel you gained knowledge that can help you score higher on the OITE? | Yes: 22/23 (96%) |
| Q2) As a result of completing Orthobullets, do you feel you gained knowledge that can help you score higher on the OITE? | Yes: 23/23 (100%) |
| Q3) As a result of completing JBJS Clinical Classroom, do you feel you gained knowledge that can help you score higher on the OITE? | Yes: 15/23 (65%) |
| Q4) How would you rate the relevancy and effectiveness of ResStudy for improving knowledge base? (1-10, 1 being least, 10 being most) | Mean score: 8/10 |
| Q5) How would you rate the relevancy and effectiveness of Orthobullets for improving knowledge base? (1-10, 1 being least, 10 being most) | Mean score: 9/10 |
| Q6) How would you rate the relevancy and effectiveness of JBJS clinical classroom for improving knowledge base? (1-10, 1 being least, 10 being most) | Mean score: 5/10 |
| Q7) How would you rate the quality of questions of the Pre-Test compared to OITE? (1-10, 1 being least, 10 being most) | Mean score: 7/10 |
| Q8) How would you rate the quality of questions of the Post-Test compared to OITE? (1-10, 1 being least, 10 being most) | Mean score: 7/10 |
| Q9) If you could only use one resource to study for the OITE, which one would you prefer? | OB: 12/23 (52%)  RS: 11/23 (48%) |
| Q10) Rank the resources in terms of question quality (Highest quality to Lowest Quality) | \| OB, RS, CC: 11/23 (48%) \| \| --- \| \| RS, OB, CC: 10/23 (43%) \| \| RS, CC, OB: 2/23 (9%) \| |
| Q11) Which resource was the easiest to use? | OB: 12/23 (52%), RS: 10/23 (43%), CC: 1/23 (5%) |

**Supplementary Table 2:** *Survey questions 12-17:* Likes/dislikes for each learning platform obtained from resident free response are summarized. **Appendix I** lists all survey questions and possible responses.

| **Learning Platform** | **Likes** | **Dislikes** |
| --- | --- | --- |
| OrthoBullets | - Best answer explanations - Well-designed Anconeus spaced repetition tool - Use-friendly interface - Answers reference back to OB topic study material | - Only has older SAE questions - No OITE questions - Answer explanations are sometimes too long - Some case presentations are incomplete |
| ResStudy | - User-friendly interface - Similar to OITE questions - Contains Recent OITE questions - Answers have references to published literature | - No spaced repetition - Does not provide explanations for all questions |
| JBJS Clinical Classroom | - Short questions - Questions are not just multiple choice - Spaced repetition | - Not similar to OITE - Limited number of questions - Explanations are not detailed |

**Supplementary Table 3:** *Survey questions 18-22:* 2019 OITE learning platform utilization, preferences, and exam performance

| **Survey Question** | **Survey Response** |
| --- | --- |
| Q18) Which learning platform(s) did you use to study for 2019 OITE? | OB alone: 1/23 (5%)  ResStudy alone: 7/23 (30%)  CC alone: 0/23 (0%)  OB and RS: 12/23 (52%)  OB, RS, and CC: 1/23 (4%)  Other: 2/23 (9%) |
| Q19) What was your preferred learning platform to prepare for 2019 OITE? | OB alone: 8/23 (35%)  RS alone: 12/23 (52%)  OB and RS: 3/23 (13%) |
| Q20) If you selected other in previous questions, please describe what you used. | 2/23 participants did not study for the 2019 OITE examination |
| Q21) What was your 2019 OITE score within-year allopathic percentile? | Mean +/- Standard deviation: 62 +/- 27 |
| Q22) What was your 2019 OITE score overall percentile? | Mean +/- Standard deviation: 52 +/- 34 |
